# Bayesian tsunami fragility modeling considering input data uncertainty

**DOI:** 10.1007/s00477-016-1230-x

**Published:** 2016-02-18

**Authors:** Raffaele De Risi, Katsuichiro Goda, Nobuhito Mori, Tomohiro Yasuda

**Affiliations:** 1 Department of Civil Engineering, Queen’s Building University Walk, University of Bristol, Bristol, BS8 1TR UK; 2 Disaster Prevention Research Institute, Kyoto University, Kyoto, 611-0011 Japan

**Keywords:** Bayesian regression, Tsunami fragility, Logistic regression, Multinomial regression, Markov Chain Monte Carlo simulation, 2011 Tohoku earthquake

## Abstract

Empirical tsunami fragility curves are developed based on a Bayesian framework by accounting for uncertainty of input tsunami hazard data in a systematic and comprehensive manner. Three fragility modeling approaches, i.e. lognormal method, binomial logistic method, and multinomial logistic method, are considered, and are applied to extensive tsunami damage data for the 2011 Tohoku earthquake. A unique aspect of this study is that uncertainty of tsunami inundation data (i.e. input hazard data in fragility modeling) is quantified by comparing two tsunami inundation/run-up datasets (one by the Ministry of Land, Infrastructure, and Transportation of the Japanese Government and the other by the Tohoku Tsunami Joint Survey group) and is then propagated through Bayesian statistical methods to assess the effects on the tsunami fragility models. The systematic implementation of the data and methods facilitates the quantitative comparison of tsunami fragility models under different assumptions. Such comparison shows that the binomial logistic method with un-binned data is preferred among the considered models; nevertheless, further investigations related to multinomial logistic regression with un-binned data are required. Finally, the developed tsunami fragility functions are integrated with building damage-loss models to investigate the influences of different tsunami fragility curves on tsunami loss estimation. Numerical results indicate that the uncertainty of input tsunami data is not negligible (coefficient of variation of 0.25) and that neglecting the input data uncertainty leads to overestimation of the model uncertainty.

## Introduction

In the last two decades, tsunamis triggered by earthquakes were responsible for 33 % of total deaths and 35 % of total economic losses globally (Guha-Sapir et al. 2015). A reliable quantification of tsunami risk becomes increasingly important for emergency officers to manage critical infrastructures and for insurance companies to quantify the expected economic losses (Goda 2015). An accurate risk analysis encompasses reliable assessments of hazard, exposure, and vulnerability. The tsunami hazard assessment is often given in a form of inundation maps, reporting inundation depths at various locations, for different tsunami rupture scenarios (Goda et al. 2014; Fukutani et al. 2015; Goda and Song 2016). Exposure assessment identifies the elements at risk, including human, built, and natural environments in coastal areas. Finally, vulnerability is represented by fragility curves, i.e. probability of reaching or exceeding specific damage states for a given hazard intensity (Porter et al. 2007). Different methods for deriving fragility curves can be found in the literature (Rossetto et al. 2014). They can be classified into four approaches: (a) empirical methods based on statistical analysis of observed damage data; (b) judgmental methods based on expert elicitation; (c) analytical methods based on evaluation of the performance response through structural analysis; and (d) hybrid techniques by combining the preceding methods. This work focuses on the empirical methods for tsunami fragility modeling.

Empirical tsunami fragility modeling requires numerous pairs of tsunami damage observations (e.g. the number of buildings reaching or exceeding a specific damage state) and explanatory variables related to hazard and exposure. Tsunami inundation depth is widely adopted as tsunami intensity measure in developing tsunami fragility curves (Koshimura et al. 2009; Reese et al. 2011; Suppasri et al. 2011, 2013; Charvet et al. 2014a). It is important to recognize that observed tsunami intensity measures are subjected to errors. Measurement errors are always present, influenced by survey techniques, equipment, and conditions. When direct measurements are not available and inundation needs to be assessed over a vast area quickly, interpolation of measured tsunami depths at nearby locations may be considered, which inevitably introduces additional errors (both random and systematic) in the gathered tsunami data. In the context of empirical fragility modeling, uncertainty associated with input hazard data should be treated adequately, because neglecting this kind of uncertainties results in potential overestimation of model uncertainty associated with developed fragility curves (Cetin et al. 2002; Der Kiureghian 2002; Straub and Der Kiureghian 2008). In the literature, problems of implementing different sources of uncertainties in fragility functions have been tackled mainly in the field of earthquake engineering (e.g. Der Kiureghian 2002; Porter et al. 2007; Bradley 2010; Baker 2015; Jalayer et al. 2015; Lallemant et al. 2015). The general frameworks for developing robust fragility models (e.g. Bayesian statistics) help implement both inherent and epistemic uncertainties in the assessment of the statistics describing fragilities (Gardoni et al. 2007). In tsunami fragility modeling, incorporation of input data errors and uncertainty has not been explored rigorously (Tarbotton et al. 2015).

The aims of this paper are twofold. Firstly, uncertainty of input tsunami hazard parameters is evaluated by consulting with two extensive tsunami inundation/run-up datasets by the Ministry of Land, Infrastructure, and Transportation (MLIT 2014) and by the Tohoku Tsunami Joint Survey (TTJS) group (Mori et al. 2011), which were compiled after the 11th March 2011 Tohoku earthquake (Fraser et al. 2013). Secondly, the effects of propagating the input data uncertainty on tsunami fragility functions are investigated by adopting Bayesian regression methods. It is noteworthy that, in general, the empirical assessment of input inundation data uncertainty is very limited due to the lack of observed data. The 2011 Tohoku tsunami offers a unique opportunity to evaluate the accuracy and consistency of the tsunami inundation data from extensive post-event field surveys and tsunami damage inspections. The MLIT database contains more than 200,000 buildings (each data entry includes building type, location, tsunami damage level, and inundation depth) and is particularly useful for developing empirical tsunami fragility curves (Suppasri et al. 2013; Charvet et al. 2014a). The TTJS database contains more than 5000 surveyed inundation and run-up heights along the Tohoku coastline and is useful for examining the tsunami inundation/run-up characteristics at both regional and local levels. The uncertainty of tsunami inundation data is evaluated by comparing the MLIT and TTJS data, noting that this comparison is not straightforward because spatial distributions of the MLIT and TTJS data differ and conversions of height to depth data are necessary by adopting a suitable digital elevation model (DEM). A potential log-normal distribution of the observations’ error is found; such result is in line with the findings of Kim et al. (2014) given the extension of the analyzed coastline.

Among procedures for developing tsunami empirical fragility curves in the literature (Tarbotton et al. 2015), three statistical approaches are considered: (a) lognormal fragility model (Peiris 2006; Porter et al. 2007), (b) binomial logistic regression (Reese et al. 2011), and (c) multinomial logistic regression (Charvet et al. 2014a; Yazdi et al. 2015). For each method, two sets of tsunami fragility functions are developed by neglecting and considering the input data uncertainty. Innovative aspects of this work in implementing the preceding three methods are that they are based on a Bayesian framework and thus the uncertainty of input data is adequately propagated in conducting the point estimation of fragility parameters. As the complexity of the fitted models increases from the lognormal to multinomial logistic models, a Markov Chain Monte Carlo (MCMC) simulation (Cheung and Beck 2010) has been conducted for the parameter estimation. To authors’ knowledge, Bayesian regression methods are applied in this paper for the first time in assessing tsunami empirical fragilities. The systematic implementation of the data and methods facilitates the quantitative comparison of tsunami fragility assessments under different assumptions.

Moreover, developed tsunami fragility models (three methods with/without input data uncertainty) are implemented in tsunami loss estimation to investigate the impact of different tsunami fragility models on risk assessment. For this, a portfolio of wooden buildings in the Tohoku region is considered. The loss assessment results are calculated using a procedure similar to Yu et al. (2013) and are presented in a form of total economic loss as well as economic loss as a function of distance from the shoreline to evaluate the spatial variation of the impact of different fragility functions.

The paper is organized as follows. Section 2 presents the mathematical formulations of the three tsunami fragility models based on the Bayesian framework. In Sect. 3, empirical uncertainty of tsunami inundation data is evaluated based on the MLIT and TTJS databases for the 2011 Tohoku earthquake. Section 4 presents the development and systematic comparison of tsunami fragility models by considering the three statistical methods with/without input data uncertainty. The tsunami loss estimation results based on different fragility models are discussed in Sect. 5, and key conclusions are drawn in Sect. 6.

## Bayesian fragility models

The three fragility models are conventionally developed through the least squares fitting procedure for the lognormal model and through the maximum likelihood method for the binomial/multinomial logistic models. In this work, these fragility models are developed through the Bayesian method by incorporating the input data uncertainty. The Bayesian approach is problem-specific and requires a good understanding of physical nature of the problem and observations (Der Kiureghian 2002). After a brief review of the Bayesian updating formula, methods of point estimation, neglecting and considering input data uncertainty are presented.

### Bayesian estimation

Let **θ** represent fragility parameters that are to be estimated based on the observed data **D**. **θ** can be treated as a set of random variables characterized by probability distribution functions. According to the Bayesian paradigm, the distribution of **θ** can be updated as new observational information becomes available (Box and Tiao 1992):

1 $$f\left( {{\varvec{\uptheta}}\text{|}{\mathbf{D}}} \right) = c^{ - 1} \cdot L\left( {{\mathbf{D}}|{\varvec{\uptheta}}} \right) \cdot f\left( {\varvec{\uptheta}} \right)$$

where *f*(**θ**) is the prior distribution of fragility parameters and represents the information available on **θ** prior to the estimation; *L*(**D**|**θ**) is the likelihood function and represents the information contained in the observation; *f*(**θ**|**D**) is the posterior distribution describing the updated estimate of **θ**; and *c* is a normalizing factor:

2 $$c = \int {L\left( {{\mathbf{D}}|{\varvec{\uptheta}}} \right) \cdot f\left( {\varvec{\uptheta}} \right) \cdot d{\varvec{\uptheta}}}$$

The likelihood function depends on the adopted type of regression and is proportional to the conditional probability of the data **D** given the parameter **θ**; therefore, assuming that observations are independent, the likelihood is given by:

3 $$L\left( {{\mathbf{D}}|{\varvec{\uptheta}}} \right) = \prod\limits_{i = 1}^{n} {f\left( {\varvec{D}_{\varvec{i}} |{\varvec{\uptheta}}} \right)}$$

where *n* is the number of observations, ***D***_***i***_ is the *i*th observation composed by the intensity measure value and the associated damage outcomes for damage states (***D***_***i***_ = [*im*_*i*_, *n*_*i,*DS1_,…,*n*_*i,*DSk_]); and *f*(***D***_***i***_|**θ**) is the value of the likelihood for the *i*th observation given **θ**.

The likelihood function represents the key factor for the propagation of the data uncertainty in the regression. According to the total probability theorem (Jaynes 2003), for the *i*th observation, the likelihood function can be written as:

4 $$f\left( {\varvec{D}_{\varvec{i}} |{\varvec{\uptheta}}} \right) = \int_{ - \infty }^{ + \infty } {f\left( {\varvec{D}_{\varvec{i}} |\varepsilon \text{,}{\varvec{\uptheta}}} \right)} \cdot f_{i} \left( \varepsilon \right) \cdot d\varepsilon$$

where *f*_*i*_(ε) is the probability density function (pdf) of the error associated with the *i*th observation, modeled as a Gaussian distribution with zero mean and standard deviation σ_ε_. The subscript *i* indicates that σ_ε_ can be different for each observation. *f*(***D***_***i***_|ε, **θ**) is the value of likelihood for the *i*th observation given **θ** and the error associated with observation ε (i.e. the likelihood for the sum of the logarithm of *im*_*i*_ and ε). The parameters that maximize the posterior represent the solutions of the problem. In the following, this is referred to as Bayesian maximum likelihood.

### Lognormal method

In the lognormal method, exceedance probabilities for damage states are calculated and median values are plotted against a range of equally spaced bins of tsunami hazard parameter (e.g. inundation depth interval of 0.5 m). The probability of occurrence of damage is:

5 $$P\left( {DS \ge ds|h} \right) = \Phi \left( {\frac{\ln h - \ln \eta }{\beta }} \right)$$

where Φ is the standard normal distribution function, *h* is the inundation depth, η is the median, and β is the logarithmic standard deviation (or dispersion parameter). The two parameters η and β are obtained by plotting the logarithm of inundation depth versus the inverse cumulative distribution function of the exceedance probability and by performing a linear regression analysis according to the following relation:

6 $$\ln h = \ln \eta + \beta \cdot \Phi^{ - 1} \left[ {P\left( {DS \ge ds|h} \right)} \right] + \varepsilon_{R}$$

where ε_*R*_ is the term representing the regression error, which is normally distributed with zero mean and unknown standard deviation σ_*R*_. In Eq. (6), *P*(*DS* ≥ *ds*|*h*) can be obtained based on the data analysis of damage outcomes for each inundation depth bin. It follows that ln*h* is normally distributed with mean function of unknown parameters η and β, and unknown standard deviation equal to σ_*R*_.

The linear regression is generally carried out through a least squares fitting procedure by minimizing the square of residuals between empirical data and calculated values. By adopting the Bayesian regression procedure, the fundamental equation is Eq. (1) where in the specific case **θ** = [η, β, σ_*R*_]. The prior distribution can be decomposed into three marginal distributions of independent variables. In particular, as priors, uniform distributions are used for η, β, and σ_*R*_ in absence of other information. The likelihood function is:

7 $$L\left( {\eta ,\beta ,\sigma_{R} \text{|}{\mathbf{D}}} \right) = \prod\limits_{i = 1}^{n} {\frac{1}{{\sqrt {2\pi } \cdot \sigma_{R} }} \cdot \exp \left\{ { - \frac{1}{{2 \cdot \sigma_{R}^{2} }} \cdot \left[ {\ln h_{i} - \ln \eta - \beta \cdot \Phi^{ - 1} \left( {P\left( {DS \ge ds|h_{i} } \right)} \right)} \right]^{2} } \right\}}$$

The triplet of the parameters is determined by maximizing the posterior numerically over the model parameter space.

When the observations are uncertain or not free from error, additional uncertainty should be considered in the problem. In this case, Eq. (6) becomes:

8 $$\ln h + \varepsilon_{\ln h} = \ln \eta + \beta \cdot \Phi^{ - 1} \left[ {P\left( {DS \ge ds|h} \right)} \right] + \varepsilon_{R}$$

where ε_ln*h*_ is the error of observational data. According to Eq. (4), the *i*th term of the product in Eq. (7) becomes:

9 $$\int_{ - \infty }^{ + \infty } {\frac{1}{{\sqrt {2\pi } \cdot \sigma_{R} }} \cdot \exp \left\{ { - \frac{1}{{2 \cdot \sigma_{R}^{2} }} \cdot \left[ {\ln h_{i} + \epsilon_{\ln h} - \ln \eta - \beta \cdot \Phi^{ - 1} \left( {P\left( {DS \ge ds|h_{i} } \right)} \right)} \right]^{2} } \right\}} \cdot f\left( {\varepsilon_{\ln h} } \right) \cdot d\varepsilon_{\ln h}$$

where *f*(ε_ln*h*_) is the pdf of the error. The error is normally distributed with zero mean and standard deviation σ_ln*h*_. The parameter σ_ln*h*_ needs to be estimated from the analysis of the error associated with observations.

Assuming independent uniform priors, likelihood, and error distribution, the formulation can be simplified further. In fact, the likelihood function remains the same as defined in Eq. (7) where the term σ_R_ is replaced by the square root of the sum of the two variances corresponding to the regression error and data error, respectively:

10 $$\sigma_{TOT} = \sqrt {\sigma_{R}^{2} + \sigma_{\ln h}^{2} }$$

This simplification facilitates the efficient estimation of the model parameters, since it avoids the integration related to Eq. (9).

### Binomial logistic method

Logistic regression is a special case of a generalized linear model and can be used for developing empirical fragility functions based on binomial data. For each damage state, individual building damage survey results provide with a binary indication of whether the considered damage state is exceeded or not and the maximum water depth at the building site. Unlike the lognormal model, the data are not organized in bins.

Let *π*_*i*_ denote the probability that the *i*th observed building is diagnosed as attaining a specific damage state *ds*. The probability that all observed buildings are classified with *ds* is:

11 $$\prod\limits_{i = 1}^{n} {\left( {\begin{array}{*{20}c} 1 \\ {y_{i} } \\ \end{array} } \right) \cdot \pi_{i}^{{y_{i} }} \cdot \left( {1 - \pi_{i} } \right)^{{1 - y_{i} }} }$$

where *y*_*i*_ is equal to 1 if the *i*th observation falls in the examined damage state and it is zero otherwise. Therefore, Eq. (11) is the likelihood function representing the probability of observed data. The term *π* may assume different forms, such as probit, logit, and loglog (Hosmer et al. 2013). In this study, the logit function is considered:

12 $$\pi_{i} = \frac{{\exp \left( {b_{1} + b_{2} \cdot \ln h_{i} } \right)}}{{1 + \exp \left( {b_{1} + b_{2} \cdot \ln h_{i} } \right)}}$$

where *b*_1_ and *b*_2_ are the model parameters. In a non-Bayesian framework, the point estimation is carried out through the maximum likelihood procedure.

When a Bayesian regression is carried out, the posterior of the model parameters can be formulated as Eq. (1), where **θ** = [*b*_1_, *b*_2_]. The prior distribution can be decomposed into two marginal distributions of independent variables. As priors, uniform distributions are used for *b*_1_ and *b*_2_ in absence of other information. The likelihood function is defined in Eq. (11). By maximizing the posterior, the model parameters can be determined numerically. An advantage of the Bayesian regression is that uncertainty of the data can be incorporated in the parameter estimation. More specifically, the *i*th term of the likelihood function becomes [see Eq. (4)]:

13 $$\int_{ - \infty }^{ + \infty } {\left( {\begin{array}{*{20}c} 1 \\ {y_{i} } \\ \end{array} } \right) \cdot \left[ {\frac{{\exp \left( {b_{1} + b_{2} \cdot \left( {\ln h_{i} + \varepsilon_{\ln h} } \right)} \right)}}{{1 + \exp \left( {b_{1} + b_{2} \cdot \left( {\ln h_{i} + \varepsilon_{\ln h} } \right)} \right)}}} \right]^{{y_{i} }} \cdot \left[ {1 - \frac{{\exp \left( {b_{1} + b_{2} \cdot \left( {\ln h_{i} + \varepsilon_{\ln h} } \right)} \right)}}{{1 + \exp \left( {b_{1} + b_{2} \cdot \left( {\ln h_{i} + \varepsilon_{\ln h} } \right)} \right)}}} \right]^{{1 - y_{i} }} } \cdot f\left( {\varepsilon_{\ln h} } \right) \cdot d\varepsilon_{\ln h}$$

A pair of parameters that maximize the posterior represents the solution of the problem.

### Multinomial logistic method

Multinomial regression is a generalized linear model that allows considering more than two outcomes at the same time and can be employed to develop empirical fragility models for multiple damage states. Charvet et al. (2014a) applied the procedure by considering binned data. More specifically, denoting the probability that structures corresponding to the *i*th observation data bin fall in the *j*th damage state *ds*_*j*_ by *π*_*ij*_, the probability that all buildings of the *i*th bin fall in the respective damage state class is given by the multinomial probability distribution:

14 $$\frac{{m_{i} !}}{{\prod\nolimits_{j = 1}^{k} {y_{ij} !} }}\prod\limits_{j = 1}^{k} {\pi_{ij}^{{y_{ij} }} }$$

where *m*_*i*_ is the total number of structures composing the *i*th observation bin, *k* is the number of damage states, and *y*_*ij*_ is the number of structures for the *i*th observation bin attaining the specific damage state *ds*_*j*_. In Eq. (14), *π*_*ij*_ may take different functions (e.g. probit, logit, and log–log) and may assume different features (e.g. nominal, ordinal, and hierarchical), as described in detail in McCullagh and Nelder (1989). In the following, the logit function is considered, and the hierarchical partially-ordered approach is adopted for the regression. In the hierarchical method, *π*_*ij*_ assumes the following form:

15 $$\pi_{ij} = \frac{{\exp \left( {b_{1,j} + b_{2,j} \cdot \ln h_{i} } \right)}}{{1 + \exp \left( {b_{1,j} + b_{2,j} \cdot \ln h_{i} } \right)}} \cdot \left( {1 - \sum\limits_{l = 1}^{j - 1} {\pi_{il} } } \right)$$

where *b*_1*,j*_ and *b*_2*,j*_ are the model parameters (i.e. the intercept and the slope of the generalized linear model, respectively); the approach is partially-ordered because the model parameters are different for all the considered damage states. Considering *k* damage states, it is possible to write *k*-1 sets of Eq. (15). The first equation presents only the fraction (i.e. no term in parenthesis) and Eq. (15) for the *k*th damage state is equal to one. In a non-Bayesian method, point estimates of the model parameters are calculated, in accordance with the maximum likelihood approach, by computing the first and second derivatives of the likelihood (or log-likelihood) function that is expressed as follows:

16 $$\prod\limits_{i = 1}^{n} {\prod\limits_{j = 1}^{k} {\pi_{ij}^{{y_{ij} }} } }$$

where *n* is the number of bins.

The preceding problem can be evaluated within a Bayesian framework, based on Eq. (1) with **θ** = [*b*_1,1_, *b*_2,1_,…,*b*_1*,k*-1_, *b*_2*,k*−1_]. The prior distribution can be composed of 2 × (*k*-1) marginal uniform distributions of independent variables. The likelihood function is given in Eq. (16). The Bayesian parameter estimation can be carried out by maximizing the posterior. Given a high number of model parameters, the parameter estimation is numerically achieved through a MCMC simulation. The Bayesian regression facilitates the inclusion of the data uncertainty. In such cases, the the *i*th term of the likelihood function becomes:

17 $$\prod\limits_{j = 1}^{k} {\int_{ - \infty }^{ + \infty } {\frac{{\exp \left( {b_{1,j} + b_{2,j} \cdot \left( {\ln h + \varepsilon_{\ln h} } \right)} \right)}}{{1 + \exp \left( {b_{1,j} + b_{2,j} \cdot \left( {\ln h + \varepsilon_{\ln h} } \right)} \right)}} \cdot \left( {1 - \sum\limits_{l = 1}^{j - 1} {\pi_{il} } } \right) \cdot f\left( {\varepsilon_{\ln h} } \right) \cdot d\varepsilon_{\ln h} } }$$

## Uncertainty of tsunami inundation data

### MLIT database

**Fig. 1:**
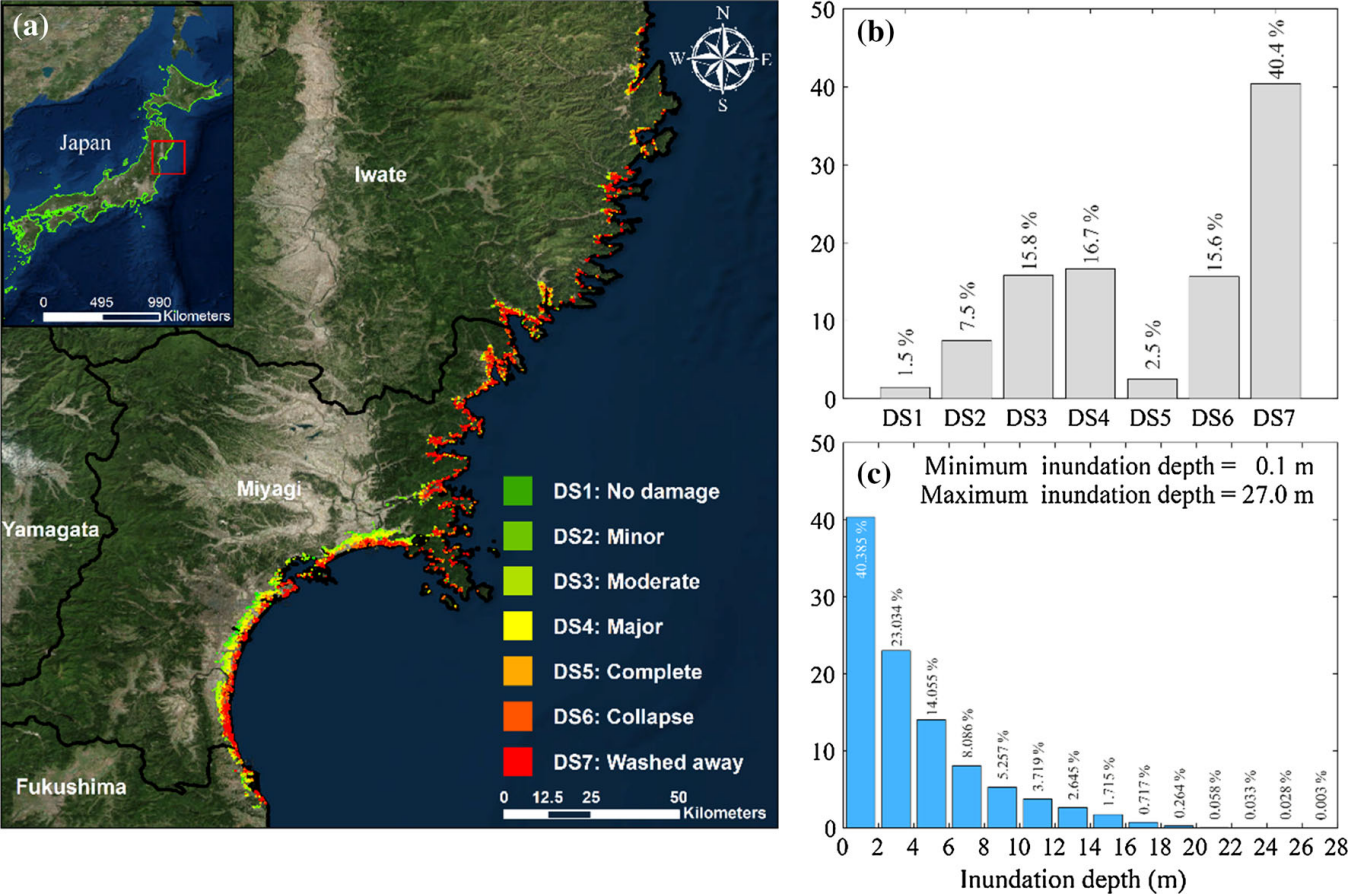
**a** Spatial distribution of surveyed wooden buildings having different damage states, **b** histogram of the tsunami damage states, and **c** histogram of the inundation depths

The input data for developing tsunami fragility models are obtained from the MLIT damage database. Buildings located in the inundated areas during the 2011 Tohoku earthquake are included in the database and are characterized by various attributes, such as geographical location, structural material, story number, tsunami inundation depth, and damage level. More than 200,000 structures are located in Iwate, Miyagi and Fukushima prefectures (from North to South). Among them, 83.9 % of the structures (176,215 buildings) have information about structural material, inundation depth, and damage state (which is necessary for developing fragility functions). The majority of the surveyed buildings (83.8 %) are wooden structures, followed by masonry structures (8.8 %), steel structures (5.0 %), and reinforced concrete (RC) structures (2.4 %). In this study, only wooden structures are considered for tsunami fragility modeling due to statistical stability. The original MLIT database adopts the tsunami damage scale with seven discrete states, namely no damage (DS1), minor damage (DS2), moderate damage (DS3), major damage (DS4), complete damage (DS5), collapse (DS6), and wash-away (DS7). Figure 1a shows the locations of the surveyed wooden buildings along about 300 km coastline near the epicenter, depicting the geographical distribution of damage states. In terms of sustained damage levels, only 1.5 % of the surveyed buildings did not suffer any damage, while 40.4 % of the buildings were washed away. The statistics of the damage levels are presented in Fig. 1b. Figure 1c shows a histogram of inundation depths that were experienced by the surveyed buildings ranging between 0.1 m and 27.0 m. Prior to tsunami fragility modeling, the original damage data are modified by combining DS6 and DS7 data, noting that these two damage states are two different descriptions of a collapse mode. Therefore, as also suggested in Charvet et al. (2014b), the seven damage states (DS1-DS7) are reduced to six (DS1-DS6/7) in the tsunami fragility analysis in the following.

The inundation depth data of the MLIT damage database are assigned based on the MLIT 100-m mesh inundation data (a separate database developed by the MLIT); in the MLIT damage database, local variations of elevation at sub-mesh levels are taken into account based on the DEM data that are developed by the Geospatial Information Authority of Japan (GSI). The GSI-DEM data are obtained by airborne laser scanner surveys and have 5-m resolution; the vertical accuracy is plus/minus 0.3 m in terms of standard deviation. The MLIT 100-m data contain information of locations, elevation, inundation depth, and inundation height. The original inundation depth data were obtained by conducting surveys of tsunami marks and interviews to local residents and from existing reports/data. When direct observations were not available, interpolation of inundation depths/heights at nearby locations was carried out. In other words, the MLIT inundation data, which are used as input tsunami hazard parameters in the fragility analysis (e.g. Suppasri et al. 2013; Charvet et al. 2014a), are subjected to errors and uncertainty.

### TTJS database

The TTJS database, which was developed independently from the MLIT database, contains post-event surveyed tsunami inundation/run-up heights along the Tohoku coast. Heights of watermarks on buildings, trees, and walls were measured using a laser range finder, a level survey, a real-time kinematic global positioning system (RTK-GPS) receiver with a cellular transmitter, and total stations (Mori et al. 2011). Generally, the accuracy of the measurements is within a few centimeters vertically. The database includes information of location (latitude and longitude as well as address), measurement date/time (used for tidal level corrections), tsunami heights, run-up distance from shoreline, tidal levels, reliability of measurements, and target objects/marks. The TTJS inundation height data can be adopted as a benchmark in assessing the errors/uncertainty associated with the MLIT inundation data as they may be considered to be more accurate (or controlled); only TTJS data with high reliability (Rank A) are used in this study. Based on the TTJS height data, the corresponding depth data can be obtained by using the GSI-DEM data (note: the correction potentially introduces systematic errors).

### Uncertainty of tsunami inundation data

The MLIT inundation depth data (as in the MLIT damage database) are subjected to two sources of uncertainty: (i) they are based on the MLIT 100-m data (i.e. errors due to interpolation/smoothing) and (ii) elevation data at the building sites are not available (thus the MLIT depth data cannot be converted to height deterministically). In other words, it is not straightforward to evaluate the accuracy of the MLIT 100-m data (both depth and height are available) quantitatively, because the data are only available at 100-m resolution (coarse for detailed assessment) and the locations corresponding to the representative values for the meshes are unknown. By taking into account the characteristics of the available data, uncertainty associated with the tsunami inundation data is assessed by comparing the MLIT versus TTJS inundation height data.

**Fig. 2:**
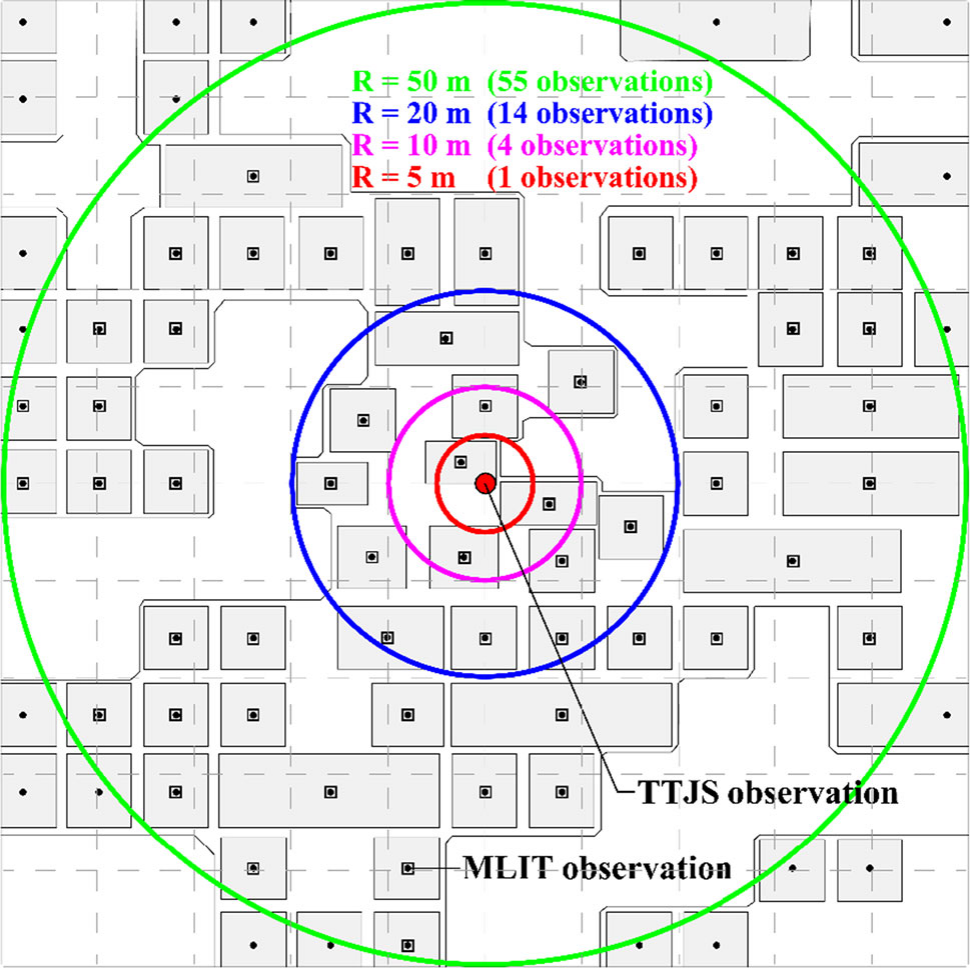
Schematic representation of the MLIT and TTJS observation points

**Fig. 3:**
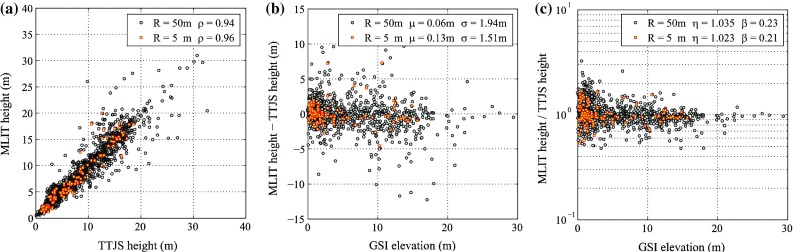
Comparison of the TTJS and MLIT inundation height data for radii of 5 and 50 m: **a** scatter plot of the TTJS and MLIT inundation height data, **b** difference between the TTJS and MLIT inundation height data with respect to the GSI-elevation, and **c** ratio between the TTJS and MLIT inundation height data with respect to the GSI elevation

To relate the MLIT data points with the TTJS data points (which have different spatial distributions and coverage), several distance radii between 5 and 50 m are considered (i.e. 5, 10, 20, and 50 m). A schematic representation of the MLIT and TTJS observation points is given in Fig. 2. The radius of 5 m is suitable for a lower bound because the baseline GSI-DEM data for the MLIT database are at this resolution. The radius of 50 m (a half of 100 m) is considered as an upper bound. With the increase of the radius, the number of data pairs increases because more points in the TTJS and MLIT databases can be associated each other (although the similarity of the selected data decreases with the separation distance due to the changes of the elevation and the local variability of the tsunami waves). Figure 3a shows the scatter plots of the MLIT and TTJS height data for the radii of 5 and 50 m. Figure 3b presents the differences between the TTJS and MLIT height data as a function of GSI elevation, while Fig. 3c displays the ratios between the TTJS and MLIT height data as a function of GSI elevation.

**Fig. 4:**
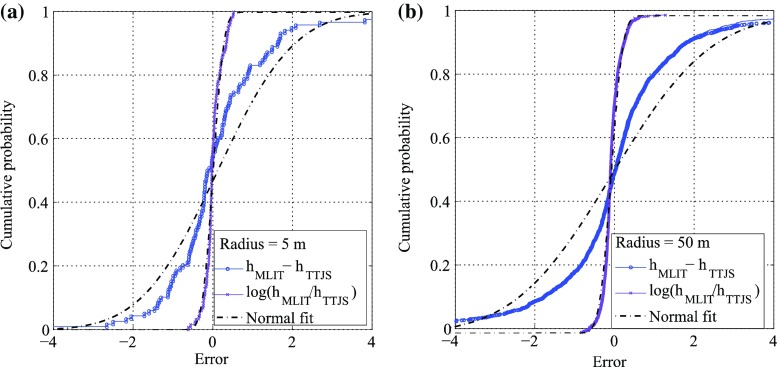
Empirical distributions of the differences (*blue dotted line*) and of the logarithmic ratios (*purple line*) between the MLIT and TTJS data in comparison with the normal fit (*dashed dotted black line*): **a** radius of 5 m and **b** radius of 50 m

Table 1 lists inundation height statistics for different values of radii between 5 and 50 m; the Pearson’s linear correlation coefficient (ρ) between the TTJS and MLIT 100-m data, the statistics of the differences between the TTJS and MLIT height data, and the statistics of the ratios between the TTJS and MLIT height data (in logarithm) are included. When the TTJS and MLIT data points are close (e.g. less than 5 m), the TTJS and the MLIT inundation height data are consistent. With the increase of the separation distance between the TTJS and MLIT data points, the consistency of the TTJS and the MLIT data decreases and the data points start to scatter more widely. This can be inspected from the decreasing trends of the linear correlation coefficient and the increasing trends of the standard deviation of the differences and ratios. Another important observation is that when the radius for interpolation becomes large (e.g. 50 m), the consistency of the inundation metrics is deteriorated significantly. Therefore, a caution is necessary in carrying out such interpolation. Taking the TTJS data as benchmark, the standard deviation of the differences of the height data can be assigned as 1.5–2.0 m and the corresponding logarithmic standard deviation of the ratios of the height data is 23 % (Table 1). Moreover, Fig. 4 shows the empirical distributions of the height differences and the height ratios for the TTJS and the MLIT data. It indicates that the height ratios are well fitted by a normal distribution (i.e. the ratios are log-normally distributed), while the height differences are not. Based on the preceding results, the inundation data are assumed to be log-normally distributed and the data uncertainty equal to 25 % is considered in the Bayesian tsunami fragility analysis (Sect. 4).

**Table 1:** Comparison of the TTJS and MLIT inundation height data

Radius (m)	ρ	μ_*h*MLIT−*h*TTJS_ (m)	σ_*h*MLIT−*h*TTJS_ (m)	η_log(*h*MLIT/*h*TTJS)_	β_log(*h*MLIT/*h*TTJS)_
5	0.96	0.13	1.50	1.023	0.21
10	0.94	0.21	1.83	1.035	0.21
20	0.95	0.24	1.65	1.053	0.22
50	0.94	0.06	1.94	1.035	0.23

## Tsunami fragility assessment

This paper focuses on wooden buildings damaged due to tsunami triggered by the 11th March 2011 Tohoku earthquake. In the following, empirical fragility curves are obtained with the different regression methods considering and neglecting the input data uncertainty.

### Results for lognormal method

**Fig. 5:**
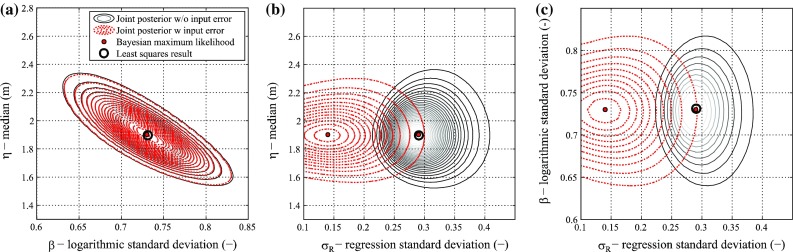
Posterior distributions of the regression parameters neglecting (*black contours*) and considering (*red contours*) the input data error for the collapse damage state (DS6/7) based on the lognormal method: **a** η−β joint distribution, **b** η−σ_*R*_ joint distribution, and **c** β−*σ*_*R*_ joint distribution

**Fig. 6:**
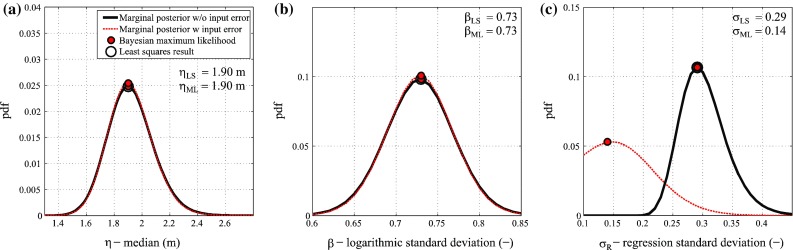
Posterior distributions of the regression parameters neglecting (*black lines*) and considering (*red lines*) the input data uncertainty for the collapse damage state (DS6/7) based on the lognormal method: **a** η marginal distribution, **b** β marginal distribution, and **c** *σ*_*R*_ marginal distribution

**Fig. 7:**
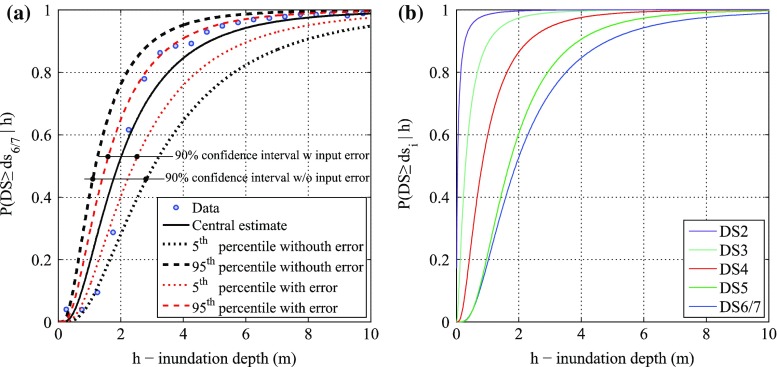
Tsunami fragility curves based on the lognormal method: **a** collapse fragility curves and 90 % confidence interval neglecting and considering input data error, and **b** tsunami fragility curves for all damage states

For the lognormal method, the inundation depth data are binned with equal intervals of 0.50 m, as suggested by Suppasri et al. (2013). Figure 5 shows the posterior joint distributions of the regression parameters (i.e. η, β, and σ_R_) without (black contours with solid line) and with (red contours with dashed line) input data errors for the collapse damage state. Figure 6 shows the corresponding marginal distributions of the three parameters for the collapse damage state. Neglecting the input data error, the Bayesian maximum likelihood estimations (i.e. red points) and the least squares results (hollow black circles) are identical. On the other hand, considering the input data error, σ_*R*_ changes significantly, showing a reduction from 0.29 to 0.14. This reduction is due to the fact that the consideration of the input data error reduces the regression error; this is expected because the term ε_ln*h*_ is moved from the left-hand side to the right-hand side in Eq. (8). The reduction in σ_*R*_ translates into a smaller confidence interval around the central estimate of the fragility function. For example, Fig. 7a shows the central estimate of the collapse (DS6/7) fragility function for wooden structures and the 90 % confidence intervals without and with input data error. Figure 7b shows the resulting fragility curves for all damage states, and Table 2 lists the parameters of the fragility curves obtained without and with input data error. The numerical results indicate that medians and logarithmic standard deviations are equal for the two considered cases, whereas the regression confidence intervals are lower when the input data error is taken into account, with a reduction of about 50 % according to available data.

**Table 2:** Parameters of fragility curves neglecting and considering the input data error based on the lognormal method

Damage state	Without input data error			With input data error		
	η (m)	β (−)	σ_R_ (−)	η (m)	β (−)	σ_R_ (−)
DS2	0.04	1.49	0.40	0.04	1.49	0.31
DS3	0.29	1.00	0.33	0.29	1.00	0.22
DS4	0.82	0.81	0.25	0.82	0.81	0.01
DS5	1.68	0.66	0.32	1.68	0.66	0.20
DS6/7	1.90	0.73	0.29	1.90	0.73	0.14

It is noteworthy that the data need to be binned (grouped) to apply this method. From a practical point of view, when the lognormal method is used by taking into account the input data error, the influence of considering the uncertainty is revealed as the narrower fragility confidence interval, whereas the central fragility curve does not change. In other words, the model dispersion is not affected.

### Results for binomial logistic method

**Fig. 8:**
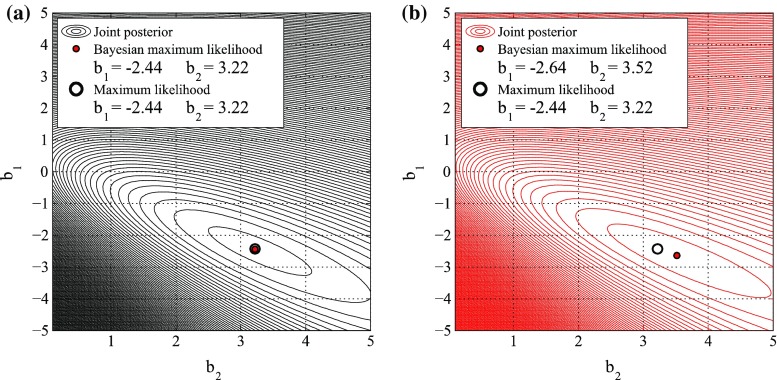
Posterior distribution of regression parameters b_1_ and b_2_ for **a** without and **b** with input data error for the collapse damage state (DS6/7) based on the binomial logistic method

**Fig. 9:**
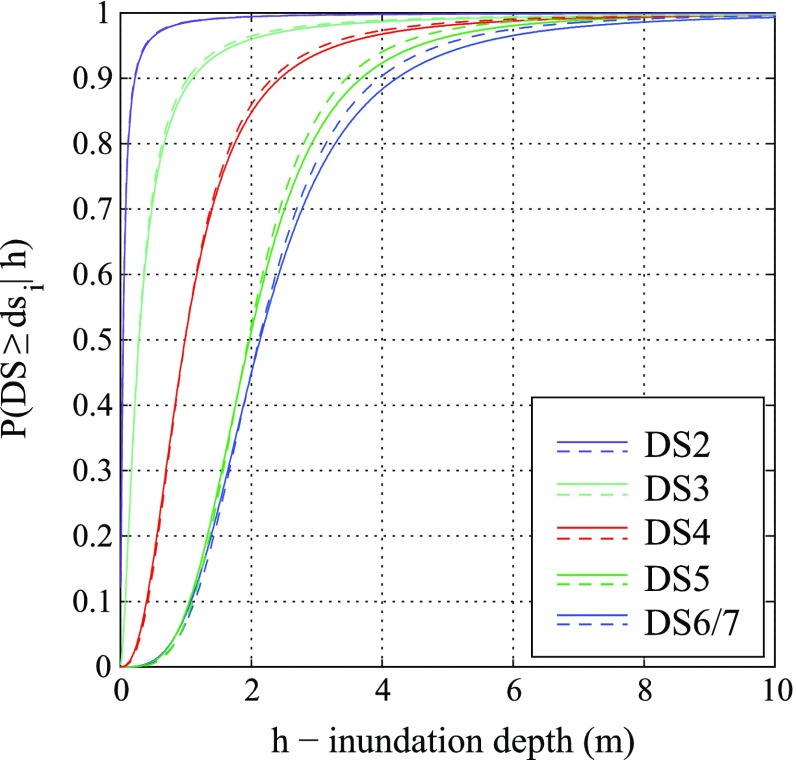
Tsunami fragility curves neglecting (*continuous line*) and considering (*dashed line*) input data error based on the binomial logistic method

For the binomial logistic method, individual records are used as un-binned data, as suggested by Reese et al. (2011). Figure 8a, b show the joint posterior distributions for DS6/7 neglecting and considering input data error, respectively. Figure 8a shows that the Bayesian maximum likelihood (red point) coincides with the maximum likelihood approach (hollow black circle). Figure 8b shows that the parameters obtained with the Bayesian and non-Bayesian procedures are different. This difference can be observed in terms of resulting fragility functions. Figure 9 shows the fragility functions for all damage states neglecting (solid line) and considering (dashed line) the input data error. Table 3 lists the numerical values of the estimated fragility parameters. The medians remain the same and the logarithmic standard deviations are decreased by about 10 % (e.g. DS5 and DS6/7).

**Table 3:** Parameters of fragility curves neglecting and considering the input data error based on the binomial logistic method

Damage state	Without input data error		With input data error	
	η (m)	β (−)	η (m)	β (−)
DS2	0.04	1.23	0.04	1.23
DS3	0.29	1.02	0.28	0.99
DS4	0.99	0.68	0.99	0.65
DS5	1.97	0.47	1.96	0.43
DS6/7	2.13	0.52	2.12	0.47

For the Bayesian binomial logistic method using ungrouped data, the effect of considering the input data error shows up in the reduced logarithmic standard deviation of a fragility curve. This means that the dispersion of the fragility model is decreased and thus more confidence on the central estimate of the tsunami fragility is achieved. It is noted that the preceding result is generally applicable to binned data (as in the multinomial logistic method in Sect. 4.3).

### Results for multinomial logistic method

**Fig. 10:**
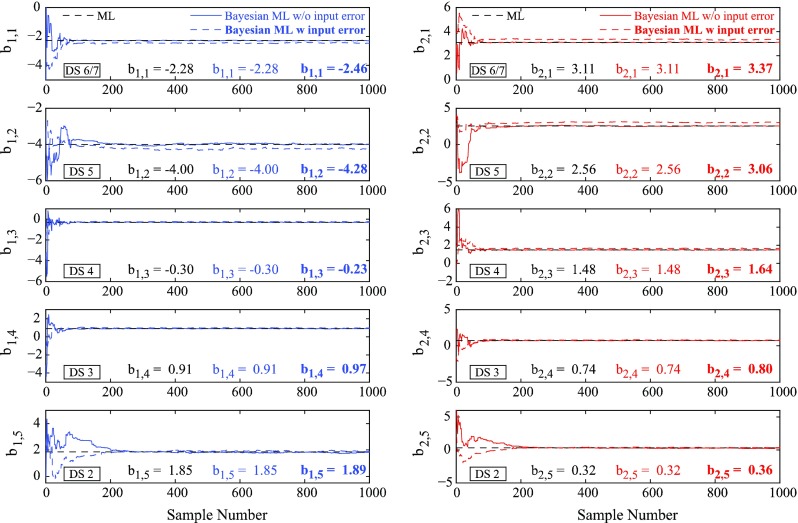
Convergence of parameter estimation based on Markov Chain Monte Carlo simulations neglecting and considering the input data error (*continuous* and *dashed line* respectively)

For the multinomial logistic method, the inundation depth data are binned with non-uniform intervals in order to obtain equally populated bins (Charvet et al. 2014a). In the estimation, the reference damage state (i.e. the damage state with probability equal to 1) is set to DS1 (Sect. 2.4); thus the order of damage states in Eq. (15) should be reversed. Figure 10 shows the results of the MCMC simulations for the Bayesian approach, neglecting and considering the input data error, represented by continuous line and dashed line, respectively. The blue and red lines are the intercept (*b*_1_) and slope (*b*_2_) of the generalized linear model, respectively. The dashed black line represents the value obtained through the maximum likelihood point estimate procedure. From the plot, the effects of the initial values of parameters take a while to disappear before the process begins to look stationary (1000 simulation steps are sufficient to achieve the convergence).

**Fig. 11:**
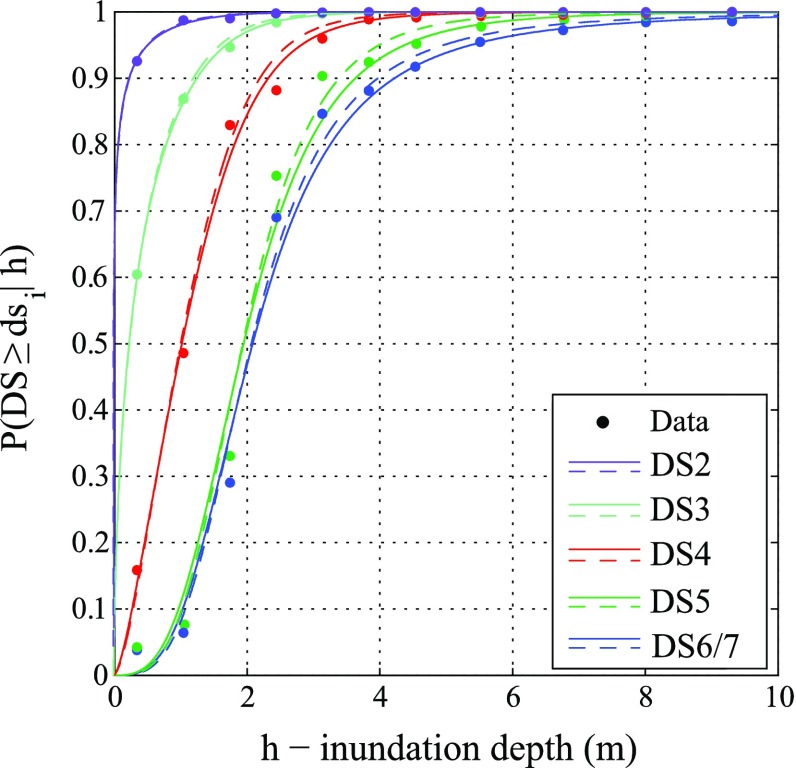
Tsunami fragility curves neglecting (*continuous line*) and considering (*dashed line*) input data error based on the multinomial logistic method

Neglecting the input data error, the final values (blue and red light numbers), obtained after 1000 simulations, converge to the value obtained with the maximum likelihood point estimate procedure (black numbers). Considering the input data error, the model parameters (blue and red bold numbers) are different with respect to the preceding case. This difference can be observed in terms of resulting empirical fragility functions, as presented in Fig. 11 neglecting (continuous lines) and considering (dashed lines) the input data error. Moreover, Table 4 lists the parameters of the obtained fragility curves for all damage states. The results show that the medians remain the same and the logarithmic standard deviations related to the case with input data errors are decreased by as large as 16 %.

**Table 4:** Parameters of fragility curves neglecting and considering the input data error based on the multinomial logistic method

Damage state	Without input data error		With input data error	
	η (m)	β (−)	η (m)	β (−)
DS2	0.01	2.50	0.01	2.38
DS3	0.23	1.92	0.24	1.61
DS4	1.02	0.83	1.01	0.77
DS5	1.95	0.51	1.95	0.45
DS6/7	2.08	0.54	2.08	0.49

Similarly to the binomial logistic method, the incorporation of input data errors into the tsunami fragility modeling only affects the model dispersion. The preceding general results are applicable to the case when un-binned data, rather than binned data, are used. This is because the multinomial logistic regression, as extension of the binomial logistic regression, can be performed for both types of data.

### Comparison of tsunami fragility models

**Fig. 12:**
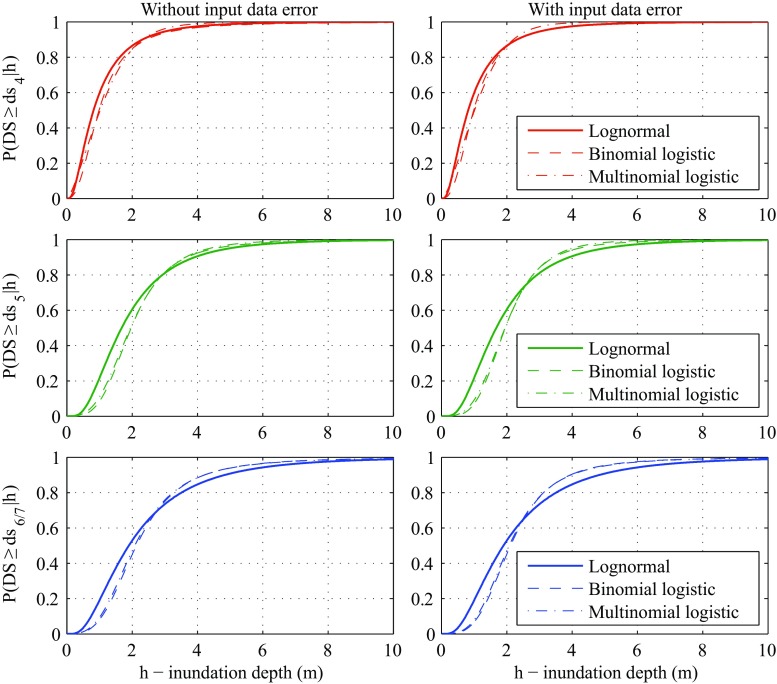
Comparison of tsunami fragility curves based on the lognormal, binomial logistic, and multinomial logistic methods for DS4, DS5, and DS6/7

The systematic applications of three Bayesian regression methods facilitate the meaningful comparisons of the tsunami fragility curves developed under different assumptions. To inspect the differences of the fragility curves visually, Fig. 12 compares the tsunami fragility curves for DS4, DS5, and DS6/7 based on the lognormal, binomial logistic, and multinomial logistic methods without and with the input data uncertainty (note: for the lognormal model, the inclusion of the input data error in the analysis has no effect on the developed fragility curve). It can be observed from Fig. 12 that medians for the lognormal method are smaller than those for the binomial/multinomial logistic method, while model dispersions for the lognormal method are greater than those for the binomial/multinomial logistic method (note: these observations are applicable to both cases neglecting and considering the input data error). This means that at low inundation depths, the fragility curves based on the lognormal method predict higher damage probabilities than those based on the binomial/multinomial logistic method. On the other hand, the opposite tendency is applicable at high inundation depths. The differences can be attributed to the forms of the base functions (lognormal versus logistic) and partly to different grouping schemes used for the three methods. The latter has relatively minor effects because the binomial and multinomial methods produce similar results although they adopt different grouping procedures.

Regarding the impact of incorporating the uncertainty of inundation depth data in the fragility modeling, the lognormal method and the binomial/multinomial logistic method affect the developed fragility models differently. When the lognormal method is adopted, only a reduction of the confidence interval can be observed, which is consistent with Cetin et al. (2002) for liquefaction triggering analysis. In contrast, when the binomial/multinomial logistic method is used, a reduction of the model dispersion is observed; the result is in accord with Der Kiureghian (2002) for seismic fragility analysis. These differences of the tsunami fragility curves caused by the different modeling approaches, have direct influence on the tsunami damage assessment (and the seismic loss estimation). In fact, for the logistic regression methods, the central estimate fragility curve obtained considering the input data error returns a greater damage probability for high values of inundation depth and a smaller damage probability for low inundation depths, in comparison with the case in which the input data error is neglected (note: the consideration of the input data uncertainty does not affect the median and thus the changing point of the increased/decreased damage probability correspond to the median inundation depth).

The differences between fragilities with and without input data uncertainty are larger for severer damage states (DS4, DS5, and DS6/7). These differences reflect the not uniform number of observations across damage states. When more observations are available, the model dispersion without input data uncertainty becomes smaller, and consequently, the effect of the input data uncertainty becomes more relevant. This is because the number of observations governs both the likelihood function and the model dispersion.

**Fig. 13:**
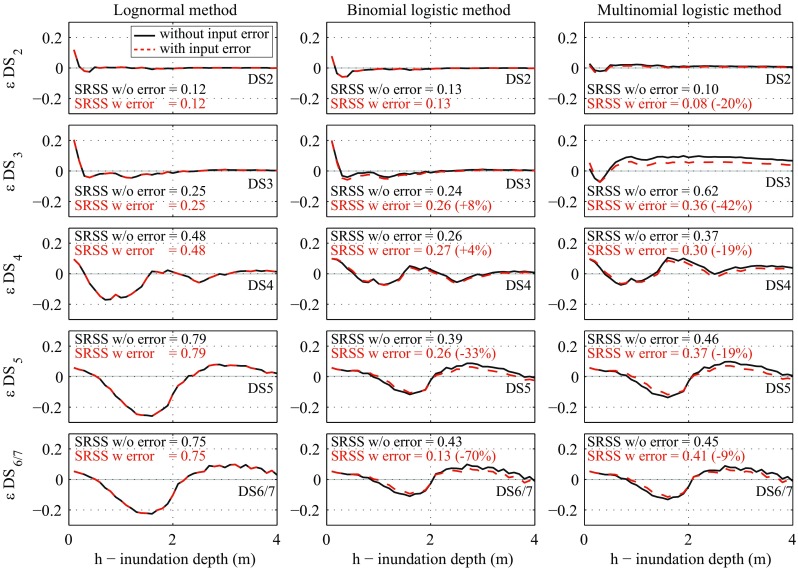
Residuals (observation minus prediction) based on the lognormal, binomial logistic, and multinomial logistic methods for all damage states neglecting (*black continuous line*) and considering (*red dashed line*) input data error

Further insights can be obtained by examining regression residuals between observed data and fitted fragility curves. Figure 13 shows the residuals (i.e. the difference between the observed data and the fitted fragility curve) obtained for each regression method; the results are presented for all damage states, neglecting and considering the input data error. To concentrate on the interval where the greatest differences occur, the residual range is focused on inundation depth between 0.1 m and 4.0 m. To discuss the differences of the observations and model predictions quantitatively, the square root of sum of squares (SRSS) of the residuals for inundation depth between 0.1 and 27.0 m is evaluated for the three methods and the calculated values are presented in the figure (note: the percentages in parenthesis show the differences between the cases without and with input data error). The results shown in Fig. 13 suggest that the profiles of the residual variations in terms of inundation depth for the three methods are similar although the magnitudes of the deviations from zero residual line for the binomial/multinomial logistic methods are smaller than those for the lognormal method (these trends can be also inspected by comparing the SRSS values). In terms of the inclusion/exclusion of the input data uncertainty in the regression analysis, (*i*) no changes are observed for the lognormal method (as expected); (*ii*) the binomial logistic method produces residuals quite similar to the case in which the input data error is neglected for DS2, DS3, and DS4, whereas it produces smaller residuals for DS5 and DS6/7; and (*iii*) for the multinomial logistic method, residuals are consistently smaller for all damage states when the input data error is taken into account in the fragility modeling. Furthermore, focusing on the SRSS values for the structural damage states (i.e. DS3, DS4, DS5, and DS6/7), the binomial logistic regression presents the smaller residuals, followed in order by the multinomial logistic regression and the lognormal regression.

It can be concluded that for the tsunami fragility data for wooden houses in the Tohoku region, using the binomial logistic method with un-binned data leads to smaller overall residuals between observed data and fitted models, and therefore is preferred with respect to the other two methods. Nevertheless, further investigations related to the multinomial logistic method are required by considering un-binned data, as this is not directly investigated in this paper.

## Tsunami loss assessment

This section presents the tsunami loss assessment according to a methodology that is similar to a performance-based earthquake engineering (PBEE) framework (Cornell et al. 2002; Goda and Song 2016). The PBEE aims to quantify the extent of damage and consequences probabilistically and is useful for assessing financial and socioeconomic impact of earthquake-related hazards (e.g. Goda 2015). Therefore, the presented results can be viewed from a decision support system perspective, which are valuable to stakeholders and decision makers.

### Set up

Empirical tsunami fragility curves based on the three different methods, considering and neglecting the input data error, can be used to carry out tsunami loss estimation. In this way, the effect induced by the input data error on the fragility curves can be further propagated to tsunami loss. The expected economic loss *E*[*L*] for a single building can be calculated as:

18 $$E\left[ L \right] = \sum\limits_{j = 1}^{k} {R_{j} \cdot \left[ {P\left( {DS \ge ds_{j} } \right) - P\left( {DS \ge ds_{j + 1} } \right)} \right]}$$

where *k* is the number of damage states; *R*_*j*_ is the repair cost associated with the damage state *j*; and *P*(*DS* ≥ *ds*_*j*_) is the exceedance probability for the damage state *j*. The buildings considered for the loss estimation are the same low-rise wooden structures (1 or 2 stories) that are contained in the MLIT database and used for the fragility modeling. Observed tsunami inundation depths at building sites are used as tsunami hazard parameter for the loss assessment. In particular, the inundation depth values are considered to be log-normally distributed with the central values corresponding to the observed ones and logarithmic standard deviation equal to 25 % (i.e. consistent with the results presented in Sect. 3.3).

Repair/replacement costs associated with damage states are computed on the basis of damage ratios 0, 5, 20, 40, 60, and 100 % for DS1, DS2, DS3, DS4, DS5, and DS6/7 (MLIT 2014), respectively. The assumed damage ratio and the replacement cost are multiplied to obtain the values of *R*. According to the MLIT (2015) and to the Japanese Construction Research Institute (CRI 2011), the mean unit construction cost is equal to 1600 $/m^2^ and the coefficient of variation is 32 %. These are the values adopted for residential low-rise wooden buildings. The unit construction cost is multiplied by the footprint area and the number of stories (information available in the MLIT database) to obtain the total construction cost for each building. The total economic loss is the sum of expected loss for all the buildings in the portfolio. The total economic loss for wooden houses is computed considering uncertainties related to the inundation depth and those related to the construction cost through a Monte Carlo simulation. In particular, 1000 simulations are performed by taking into account variability of the construction costs and the tsunami inundation depths for individual buildings.

### Total tsunami loss and risk disaggregation

Table 5 lists the central estimates of the total tsunami loss based on the three regression methods neglecting and considering the input data error. The lognormal method results in the greatest total expected loss (by about 4 %) when compared with the binomial and multinomial logistic methods. Whereas the logistic methods return similar values of total expected loss with a mean difference of 0.5 %. The major difference of the total expected loss is a consequence of the differences in the tsunami fragility curves at smaller inundation depths (Fig. 12).

**Table 5:** Expected losses with and without input data error for the three regression methods

Regression method	*E*[*L*] expected losses ($)	
	Without input data error	With input data error
Lognormal method	1.534 × 10^10^	1.534 × 10^10^
Binomial logistic method	1.465 × 10^10^	1.475 × 10^10^
Multinomial logistic method	1.475 × 10^10^	1.481 × 10^10^

The consideration of the input data uncertainty does not affect the expected total loss for the lognormal method, whereas there are slight increases of the expected tsunami loss by 0.68 and 0.43 % for the binomial logistic method and the multinomial logistic method, respectively. These numbers are small in relative terms, but they result in significant differences as actual values (i.e. hundreds of millions of dollars). They can be explained by the changes of the tsunami fragility curves (i.e. same median with reduced model dispersion). In fact, the greatest part of the exposure is located in the first 1500 m from the shoreline, where very large tsunami inundation depths were observed at many coastal cities and towns during the 2011 Tohoku tsunami (Fraser et al. 2013; Goda et al. 2014). Therefore, increased damage probabilities for collapse and complete damage, combined with greater damage ratios, result in greater tsunami loss estimates.

**Fig. 14:**
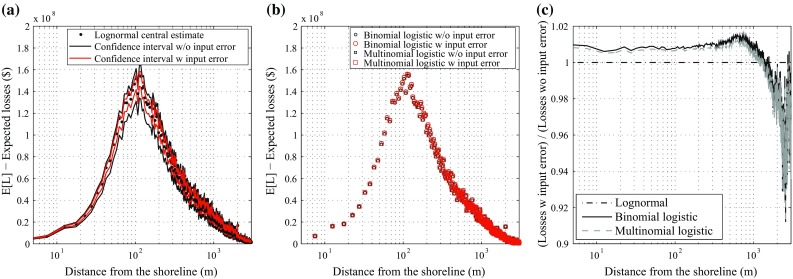
Expected tsunami losses as a function of distance from the shoreline based on the lognormal method (**a**) and the binomial and multinomial logistic methods (**b**), and **c** ratios of expected tsunami losses neglecting and considering input data error based on the three regression methods

To further investigate the spatial distribution of the tsunami loss, a risk disaggregation is shown in Fig. 14 in terms of distance from the shoreline. The disaggregation is calculated by summing up the tsunami loss with an interval of 5 m. Generally, the tsunami loss profiles for the three methods are similar; the peaks are observed at 100 m from the coastline (where a large number of buildings are located and they experience high inundation depths). The results obtained with the lognormal method (Fig. 14a) indicate that the central estimate does not change depending on how the input data error is treated in the fragility modeling (as expected from Sect. 4.1); while the 90 % confidence interval becomes narrower around the central estimate when the input data uncertainty is taken into account. The reduction of the confidence interval is approximately equal to 13 %. Figure 14b shows the results obtained with the binomial and multinomial logistic methods. Tsunami loss profiles for the two cases are similar. Finally, Fig. 14c presents the ratio between expected tsunami loss profiles obtained considering and neglecting the input data error for the different regression methods. In Fig. 14c, the result for the lognormal method is constant with a ratio of 1.0. For the logistic regression approaches, the expected tsunami loss obtained considering the input data error is greater by about 1 % with respect to the case in which the input data error is ignored. This trend is observed up to about 1500 m from the shoreline, where 90 % of the total loss exposure is concentrated.

## Summary and conclusions

This work presented a Bayesian statistical framework to consider the input data uncertainty in the empirical tsunami fragility modeling and investigated the effects of this kind of uncertainty on risk assessment for tsunamis. Three most common methods for tsunami fragility modeling were considered: the lognormal model based on linear regression, the binomial logistic regression, and the multinomial logistic regression. The developed Bayesian approaches were applied to the extensive MLIT tsunami damage data for residential wooden structures obtained from the 11th March 2011 Tohoku earthquake by neglecting and considering the input data error. To evaluate the uncertainty of tsunami inundation (i.e. input data for tsunami fragility modeling) from empirical perspectives, inundation heights available in the MLIT and TTJS databases were used. The data analysis indicated that a potential input error of 20–25 % in terms of the coefficient of variation is suitable for the Tohoku tsunami inundation data. This magnitude of the input data uncertainty was then propagated in the tsunami fragility modeling as well as tsunami loss estimation using the three regression procedures.

The systematic assessment and comparison of the tsunami fragility curves developed using different regression approaches within the Bayesian statistical framework indicated that considering the input data error leads to a reduction of the confidence interval for the lognormal method, whereas the incorporation of the input data uncertainty results in decreased model dispersion. The tsunami fragility models based on the lognormal method generally have smaller medians with greater dispersions, in comparison with those based on the binomial/multinomial logistic method. It is important to highlight that the differences in the developed fragility curves have consequential influence on the estimated tsunami loss (e.g. reduced confidence interval of the central loss estimate for the lognormal regression and increased expected tsunami loss for the logistic regressions). It was also observed that considering the input data error through the proposed Bayesian framework leads to a reduction of the residuals between observed data and fitted fragility models. Overall, the binomial logistic method may be preferred among the three regression methods, since it achieved the smaller residuals.

It is important to underline that in developing empirical tsunami fragility curves, accounting for the input data uncertainty makes their use in damage and loss assessment more reliable. In fact, such fragilities are suitable for a more general use, since the restriction of application with respect to the areas for which they are calibrated is removed (in a sense of input data uncertainty; regional dependence of the fragility models on the characteristics of local structures still exists). Further extensions and developments of this work can be considered in three aspects. One is to consider the entire posterior distributions of regression parameters in fragility modeling, in order to obtain a more robust estimation of the fragility functions. The second is to consider binned data for the binomial logistic regression and un-binned data for the multinomial logistic regression in order to facilitate the more complete and systematic comparisons of the tsunami fragility methods, going beyond the scope that the current literature concerns. The last aspect is to consider models accounting for inundation flow velocity, fluid momentum or the other relevant fluid dynamic information as explanatory hazard variable in addition to flow depth, such as the models developed by Charvet et al. (2015) for inundation flow velocity. For the latter case, the quantification of uncertainty is a real challenge given the limited amount of observed data in terms of tsunami flow velocity.
